# Glycotyping and Specific Separation of Listeria monocytogenes with a Novel Bacteriophage Protein Tool Kit

**DOI:** 10.1128/AEM.00612-20

**Published:** 2020-06-17

**Authors:** Eric T. Sumrall, Christian Röhrig, Mario Hupfeld, Lavanja Selvakumar, Jiemin Du, Matthew Dunne, Mathias Schmelcher, Yang Shen, Martin J. Loessner

**Affiliations:** a Institute of Food, Nutrition and Health, ETH Zurich, Zurich, Switzerland; INRS—Institut Armand-Frappier

**Keywords:** teichoic acids, serovar, cell wall-binding domain, bacteriophage, *Listeria*, receptor binding protein, serotyping

## Abstract

The Gram-positive pathogen Listeria monocytogenes can be subdivided into at least 12 different serovars, based on the differential expression of a set of somatic and flagellar antigens. Of note, strains belonging to serovars 1/2a, 1/2b, and 4b cause the vast majority of foodborne listeriosis cases and outbreaks. The standard protocol for serovar determination involves an agglutination method using a set of sera containing cell surface-recognizing antibodies. However, this procedure is imperfect in both precision and practicality, due to discrepancies resulting from subjective interpretation. Furthermore, the exact antigenic epitopes remain unclear, due to the preparation of the absorbed sera and the complex nature of polyvalent antibody binding. Here, we present a novel method for quantitative somatic antigen differentiation using a set of recombinant affinity proteins (cell wall-binding domains and receptor-binding proteins) derived from a collection of *Listeria* bacteriophages. These proteins enable rapid, objective, and precise identification of the different teichoic acid glycopolymer structures, which represent the *O*-antigens, and allow a near-complete differentiation. This glycotyping approach confirmed serovar designations of over 60 previously characterized *Listeria* strains. Using select phage receptor-binding proteins coupled to paramagnetic beads, we also demonstrate the ability to specifically isolate serovar 1/2 or 4b cells from a mixed culture. In addition, glycotyping led to the discovery that strains designated serovar 4e actually possess an intermediate 4b-4d teichoic acid glycosylation pattern, underpinning the high discerning power and precision of this novel technique.

**IMPORTANCE** Listeria monocytogenes is a ubiquitous opportunistic pathogen that presents a major concern to the food industry due to its propensity to cause foodborne illness. The *Listeria* genus contains 15 different serovars, with most of the variance depending on the wall-associated teichoic acid glycopolymers, which confer somatic antigenicity. Strains belonging to serovars 1/2 and 4b cause the vast majority of listeriosis cases and outbreaks, meaning that regulators, as well as the food industry itself, have an interest in rapidly identifying isolates of these particular serovars in food processing environments. Current methods for phenotypic serovar differentiation are slow and lack accuracy, and the food industry could benefit from new technologies allowing serovar-specific isolation. Therefore, the novel method described here for rapid glycotype determination could present a valuable asset to detect and control this bacterium.

## Abstract (precis)

Listeria monocytogenes is a ubiquitous opportunistic pathogen that presents a major concern to the food industry due to its propensity to cause foodborne illness. The *Listeria* genus contains 15 different serovars, with most of the variance depending on the wall-associated teichoic acid glycopolymers, which confer somatic antigenicity. Strains belonging to serovars 1/2 and 4b cause the vast majority of listeriosis cases and outbreaks, meaning that regulators, as well as the food industry itself, have an interest in rapidly identifying isolates of these particular serovars in food processing environments. Current methods for phenotypic serovar differentiation are slow and lack accuracy, and the food industry could benefit from new technologies allowing serovar-specific isolation. Therefore, the novel method described here for rapid glycotype determination could present a valuable asset to detect and control this bacterium.

## INTRODUCTION

Listeria monocytogenes is a Gram-positive, opportunistic, intracellular pathogen capable of causing severe and potentially fatal infections in susceptible individuals. This bacterium is ubiquitous in nature and able to survive in harsh environmental conditions, such as low temperature and pH (1, 2). The organism occurs widely in food, especially processed meat, poultry, seafood, dairy products, and produce, and is responsible for causing outbreaks of listeriosis. With a mortality rate of up to 30% (3), it is vital that regulatory agencies, as well as the food industry itself, have rapid tools at their disposal to quickly isolate and differentiate L. monocytogenes contaminates. South Africa recently suffered from one of the largest L. monocytogenes outbreaks to date (4), with more than 1,000 reported cases resulting in 183 fatalities, thus emphasizing the necessity for improving diagnostics and continuing research toward the control of this bacterium.

Within the genus *Listeria*, strains can be differentiated by serovar (SV), of which there are at least 15 types (5). Of the 12 SVs within L. monocytogenes (1/2a, 1/2b, 1/2c, 3a, 3b, 3c, 4a, 4b, 4c, 4d, 4e, and 7), mainly SVs 1/2a, 1/2b, and 4b are associated with disease, with SV 4b being responsible for nearly all outbreaks, despite its relative rarity (6). This is in contrast to SVs 1/2a and 1/2b, which are most commonly found in the food environment (7). SV identity is determined by structural variation of the flagellar and somatic antigens (conferred by the flagellum proteins and the cell wall teichoic acids [WTAs], respectively), with the somatic antigens representing the main diversity determinant, and primary indicator (8). WTA is the most abundant cell wall-associated glycopolymer in many Gram-positive bacteria (9). For *Listeria* bacteria, the basic structure is made up of repeating units of ribitol-phosphate (type I) or ribitol-phosphate-*N*-acetylglucosamine (type II) (10). These molecules are conjugated covalently to the *N*-acetylmuramic acid of peptidoglycan via a conserved linkage unit and extend well beyond the surface of the outermost peptidoglycan layer (11). WTAs can vary greatly through further decoration of the backbone with rhamnose, *N*-acetylglucosamine (GlcNAc), glucose, galactose, or *O*-acetylation (10). This structural and constitutional variation serves as the basis for somatic antigenicity, the primary determinant of SVs, as WTAs can serve as epitopes for distinguishing antibodies (12). Since SVs are deemed clinically relevant (13), one can theorize that the WTA structures themselves confer virulence. In support of this, it has been shown that cells typed as SVs 3 and 7 are merely mutants derived from SV 1/2 strains that have lost the GlcNAc and rhamnose WTA decorations through point mutations and are otherwise genetically identical (14). Similarly, SV 4d strains appear to be mutants derived from an SV 4b background that have lost their WTA galactose decoration (15). These findings suggested that the decorations themselves contribute to virulence since SVs 3, 7, and 4d are not known to cause listeriosis. Indeed, we and others have recently shown that these galactose and rhamnose decorations contribute directly to phage susceptibility, resistance to osmotic pressure, growth fitness, and the maintenance of major virulence factors, such as InlB and Ami (15–19). These revelations are now leading to a renewed focus on SV identity and show that phenotypic evaluations may hold more merit in certain situations over whole-genome sequencing (WGS), which otherwise offers far greater discerning power.

SV determination using the classical slide agglutination test has severe limitations because of the inadequate availability of the required antibodies, wide discrepancies due to antisera preparation, and the subjective nature of visual determination of agglutination patterns. Furthermore, the antisera reactions or patterns (8, 20) cannot be directly correlated to known WTA backbone structures or glycosylation patterns, meaning that the recognized epitopes themselves remain vague. SV identification via PCR presents a clearer method for such purposes (21). However, given that a shift from SV 4b to 4d or SV 1/2 to 3 results from single nucleotide polymorphisms (SNPs) in glycosyltransferases (14, 15) and the template DNA can stem from inactivated bacteria, PCR also presents significant shortcomings.

The inherent ability of bacteriophages (phages) to bind and infect a limited host range of bacteria is largely dependent on specific recognition by phage-encoded affinity proteins, which provide ideal tools for development as bacterial diagnostics (22). Most phages feature a complex baseplate structure at their tail’s distal end that coordinates host adsorption and phage DNA injection (23). Attached to the baseplate are dedicated receptor binding proteins (RBPs), typically identified as tail fibers or globular tail spikes, that mediate binding of the phage particle to host-specific receptors. Phage RBPs demonstrate highly specific host binding ranges that have led to their development as bio-probes for other bacterial detection assays, such as for *Salmonella* spp. (24, 25) and *Shigella* spp. (26). Conveniently and pertinently, all L. monocytogenes phages with known receptors recognize different WTA structural patterns during adsorption to their hosts (27–29), correlating with the fact that they bind and infect *Listeria* strains in an SV-dependent manner (30–32). This has led to the development of various tools using *Listeria* phages and their proteins for typing and detection of *Listeria* (31, 33). Phage RBPs can make ideal lectin-like proteins for binding SV-specific WTA structures (28). Similarly, *Listeria* phage-encoded endolysins, which lyse the peptidoglycan at the end of the lytic reproduction cycle, possess cell wall-binding domains (CBDs) at their C termini, which either directly recognize WTA or bind peptidoglycans in a WTA-dependent manner. Again, due to their strict specificity and high binding affinity, these proteins are ideal tools for rapid and specific detection of *Listeria* (10, 34–37).

Using a large library of recombinant phage RBPs and endolysin CBDs, we developed a novel glycotyping scheme for specific differentiation of *Listeria* strains with greater somatic antigen discerning power than serotyping. Here, we present a unique fluorescence-based approach for serovar profiling, allowing for the quantification of cell wall binding, thus creating a unique phenotypic fingerprint. Furthermore, we demonstrate the ability to isolate strains belonging to the pathogenic SVs 1/2 and 4b from a mixed culture of *Listeria* using WTA-recognizing phage proteins coupled to paramagnetic beads, essentially allowing for rapid isolation and enrichment based entirely on the presence of virulence-associated epitopes.

## RESULTS

**FIG 1:**
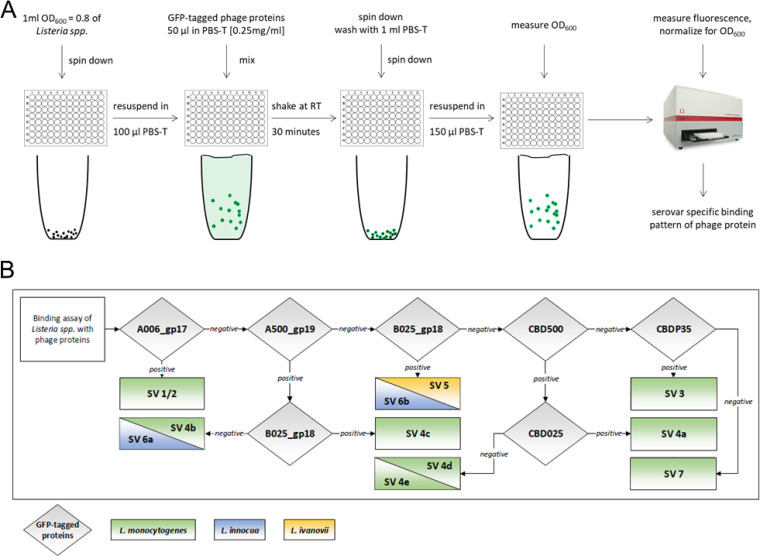
*Listeria* glycotyping using fluorescent protein-tagged phage proteins. (A) Workflow for the determination of serotype-specific binding patterns of GFP-tagged phage proteins. Each GFP-tagged phage protein is individually incubated with *Listeria* cells and fluorescence measured after removal of unbound protein and normalized to cell density (OD_600_). (B) Scheme used for sequential glycotype and serovar determination of L. monocytogenes strains based on binding patterns obtained through the procedure shown in A.

Bacteria from log-phase cultures (optical density at 600 nm [OD_600_] of ∼0.8) were incubated with each of six different GFP-tagged phage affinity proteins and thoroughly washed, and the fluorescence was determined using a spectrophotometer, a schematic for which is outlined in Fig. 1A. The glycotyping readout represents a unique and quantitative fingerprint, which provides an objective means of determining the presence or absence of SV-defining WTA structures. Overall, the rapid assay takes only 30 min, from harvesting the cells to evaluation of results.

### Selection of phage proteins for glycotyping of *Listeria* strains.

The assay includes three phage endolysin CBDs, namely, CBD025 (34), CBDP35 (34), and CBD500 (37), all of which have been previously validated as effective probes for the differentiation of *Listeria*. To identify suitable phage RBPs for complementing the glycotyping scheme, putative baseplate-associated proteins of various *Listeria* phages with distinct host ranges were subjected to *in silico* analyses using HHpred (38), BLASTp (39), and Jpred secondary structure predictions (40). Based on the conserved synteny of *Listeria* siphoviral structural genes (23), four tail spike RBPs from bacteriophages A006 (gp17), A500 (gp19) (16), PSA (gp15) (41), and B025 (gp18) were selected to expand the repertoire of GFP-tagged phage proteins, aiming at SV-specific glycotype identification. We produced and purified recombinant His-tagged versions of these proteins (see Fig. S1 in the supplemental material) and examined their specific binding patterns (Table 1) by testing strains of each SV with known WTA structures (10) (see Fig. S2 in the supplemental material). Confirmation of binding positivity via fluorescence microscopy for all seven phage proteins (see Fig. S3 in the supplemental material) enabled us to design a scheme for the systematic identification of an unknown *Listeria* strain, based on the quantitative fluorescence signal from only six of the GFP-tagged affinity proteins bound to the bacterial surface (Fig. 1B).

**TABLE 1:** Binding patterns of phage proteins to *Listeria* strains with known serovars^*a*^

Protein	Binding pattern by serovar or species											
	1/2a	3a	7	4a	4b	4c	4d	4e	5	6a	6b	S. aureus
GFP												
A500_gp19					+	+		∼		+		
A006_gp17	+											
PSA_gp15					+			∼		+		
B025_gp18						+			+		+	
CBD500				+	+	+	+	+	+	+	+	
CBD025				+					+		+	
CBDP35	+	+		+	+/−	+	+/−	+/−	+	+/−	+	+/−

a The glycotype fingerprint for each *Listeria* serovar is presented, with the phage binding proteins listed on the left. +, positive binding above the quantitatively determined threshold; +/−, binding can vary depending on the strain; ∼, weak binding.

### The phage RBP A006_gp17 recognizes SV 1/2 WTA with high selectivity and sensitivity.

**FIG 2:**
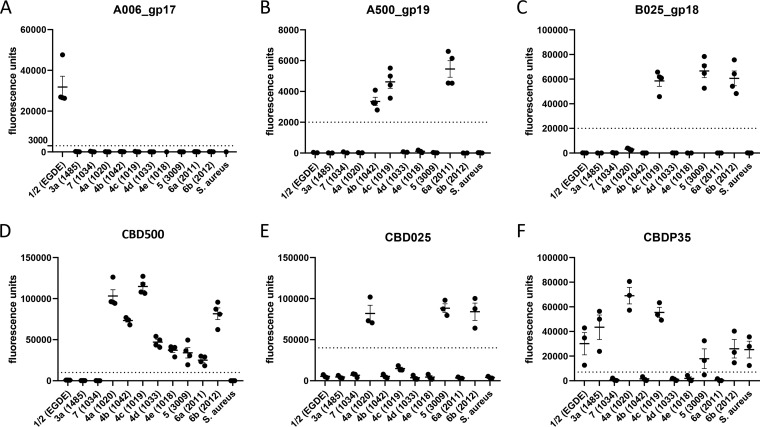
Quantitative determination of GFP-tagged phage protein binding to *Listeria* strains representing different serovars and glycotypes. (A to F) Fluorescence-based quantification of binding of the indicated proteins to different *Listeria* cell surfaces (strain code in parentheses). Staphylococcus aureus was used as an internal control (see the text). The dotted line represents the threshold/cutoff value, above which the fluorescence (presented as arbitrary fluorescence units; *y* axis) indicates positive binding for a given strain. Measurements were performed at least in triplicates; error bars represent standard error of the mean (SEM).

A terminal rhamnose decoration on the ribitol-phosphate backbone of type I WTA is the defining feature of strains belonging to SV 1/2a, 1/2b, and 1/2c (see Fig. S2). While the RBP from phage A118 has previously been shown to recognize this molecule (28), we found that its binding affinity is relatively low and not adequate for our purpose. Alternatively, phage A006 possesses an SV 1/2-specific host range (42), suggesting that it may also adsorb to rhamnosylated type I WTA. *In silico* analyses suggested that A006_gp17 features three distinct domains, with its C terminus (Ile639-Asp720) structurally resembling the receptor-binding head domains of RBPs from lactococcal phages p2 (PDB identifier [ID]: 1ZRU [43]) and TP901-1 (PDB ID: 2FOC [44]). We expressed and purified an N-terminal truncation of A006_gp17 (Met346-Asp720) and the full-length protein, both tagged with an N-terminal GFP. Both constructs demonstrated a high affinity for the cell walls of several SV 1/2 strains (see Fig. S4 in the supplemental material), including the archetype SV 1/2a strain EGDe. Since it produced a higher relative fluorescence intensity and was more readily expressed than the full-length construct, the truncated version (designated herein as A006_gp17) was selected for SV 1/2 strain identification (Fig. S4) and is clearly specific for SV 1/2 (Fig. 2A).

### The RBPs of phages A500 and B025 identify SVs 4b, 4c, 5, 6a, and 6b.

The ability of bacteriophage A500 to bind to *Listeria* host cells requires galactosylated type II WTA, meaning that SV 4b, 4c, and 6a strains can be infected (15, 29). Based on A500 genomics, both A500_gp19 (342 amino acids [aa]) and A500_gp20 were predicted to encode the putative RBPs. Despite their similar size, *in silico* analysis suggested that these putative tail spikes have different architectures. Gp19 structural predictions showed strong relatedness to an assortment of phage tail spikes, including Acinetobacter phage phiAB6 (PDB ID: 5JS4) (45) and *Salmonella* phage Det7 (PDB ID: 6F7D) (46). As expected, GFP-tagged A500_gp19 bound only to SV 4b, 4c, and 6a strains, all of which feature galactosylated WTA (see Fig. S2). Therefore, A500_gp19 was implemented into the typing scheme for SV 4b, 4c, and 6a identification (Fig. 1B and 2B). Phage B025 possesses an interesting host range since it may infect SV 4c, 5, and 6b strains (31, 47). We hypothesized that its putative RBP may be encoded by *orf18*, based on its location two genes downstream of the putative tape measure protein (*orf14*), equivalent to the position of tail spike genes of phages PSA and A500 (47). The binding range of recombinant GFP-tagged gp18 was found to nicely correlate with its parent phage host range (Fig. 2C). Thus, we introduced B025_gp18 into the glycotyping scheme for the identification of WTA structures specific to SVs 4c, 5, and 6b (Fig. 1B).

### Identification of serovars 3, 4a, 4d, 4e, and 7 using endolysin CBDs.

The ligands targeted by the endolysin cell wall-binding domains CBDP35, CBD500, and CBD025 have previously been studied (10). In short, CBDP35 recognizes the unsubstituted GlcNAc moiety in the WTA type I of SVs 1/2, 3, and 4a; CBD500 recognizes the *O*-acetylated integrated GlcNAc residue typical for WTA type II strains (SVs 4a/b/c/d/e, 5, and 6a/b); and CBD025 exclusively recognizes GlcNAc conjugated to the WTA’s ribitol backbone at the carbon position C2 instead of C4, thus conferring specificity for SVs 4a, 4c, 5, and 6b. Because Staphylococcus aureus Newman also harbors a GlcNAc in its WTA polymer (48), it is also recognized by CBDP35 and can, therefore, be used as an internal control. Evaluating the cell wall binding of GFP-fused constructs confirmed that fluorescence quantification allows for clear glycotype and SV discrimination (Fig. 2D, E, and F). Despite CBDP35 and CBD500 demonstrating a certain level of binding variability across SVs, we defined suitable cutoffs indicating positive binding for CBDP35, CBD500, and CBD025 for facilitating determination according to the scheme shown in Fig. 1B.

### Glycotyping correctly identifies a comprehensive selection of *Listeria* strains.

We next wanted to test the applicability of our quantitative *Listeria* glycotyping procedure using a cohort of more than 60 *Listeria* strains, whose SV designations either are known from the literature or have been determined using the traditional agglutination serotyping method (see Table S1 in the supplemental material). For 58 of the 60 tested strains, the measured fluorescence intensities matched the expected cutoffs for positive or negative binding, and our algorithm determined the SVs correctly, thus demonstrating the reliability and experimental robustness of the system. Notably, SV 1/2* strain 1442, which features a single nucleotide mutation in a glycosylation gene, resulting in a lack of GlcNAc decoration and CBDP35 binding (36), was labeled by GFP-tagged A006_gp17, thus presenting a novel fingerprint which can only be identified by glycotyping. In contrast, conventional serotyping does not allow for the differentiation of such SV 1/2* strains (14), highlighting the advantage of glycotyping as presented here. Moreover, the previously published SV 3 strain ATCC 19113 (WSLC 1002) and SV 4b strain ATCC 13932 (WSLC 1039) surprisingly revealed binding patterns typical for SV 1/2 and SV 4d/4e, respectively, and were subsequently reserotyped, which confirmed the glycotyping finding, suggesting they had been previously incorrectly designated. Thus, we demonstrate the concept for successful SV differentiation within the *Listeria* genus by glycotyping and provide two examples of successful reevaluation.

### RBP-coated paramagnetic beads for differential separation of SV 1/2 and 4b cells.

The SV patterns of L. monocytogenes correlate with virulence, with SVs 1/2 and 4b commonly identified as the causative agents for the majority of human listeriosis cases (13). To further increase the robustness of the system and provide a greater level of differentiation for SV 4b strains, we evaluated the tail spike RBP (gp15) of the SV 4b-specific phage PSA, whose crystal structure has recently been solved (41). Of note, this protein was not necessary for the typing panel, but its high specificity was presumed to be useful for differential separation. A recombinant GFP-tagged version of its C-terminal 173 residues (designated here as PSA_gp15) possesses a narrow binding range, by recognizing the cell walls of only SV 4b and 6a strains (Table 1), which both feature the same Gal decoration at the same relative position on the monomer, despite being from different species.

**FIG 3:**
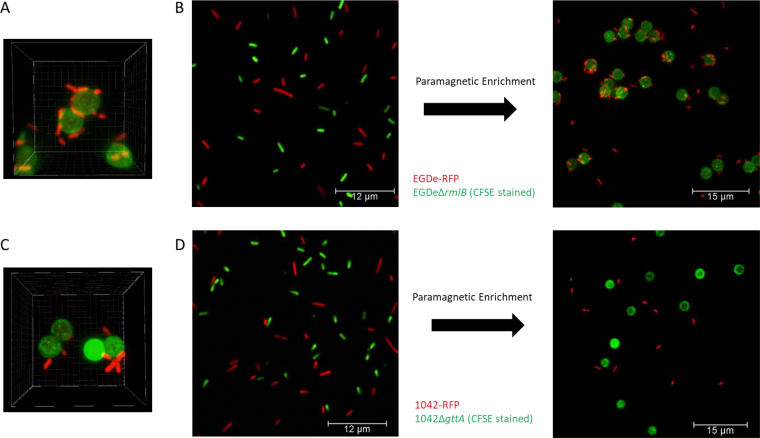
Serovar and glycotype-specific separation of *Listeria* cells using A006_gp17- and PSA_gp15-coated paramagnetic beads, respectively. (A) Three-dimensional rendering of A006_gp17-GFP coupled to epoxy-coated magnetic beads after incubation with EGDe::pPL2(*rfp*) and imaged using fluorescence confocal microscopy. (B) Equal 1:1 mixture of EGDe::pPL2(*rfp*) (SV 1/2) and EGDeΔ*rmlB* (SV 3a, CFSE-stained) before and after enrichment using paramagnetic beads coated with A006_gp17-GFP. (C) Three-dimensional rendering (same as A) of PSA_gp15-GFP coupled to epoxy beads after incubation with 1042::pPL2(*rfp*). (D) Equal 1:1 mixture of 1042::pPL2(*rfp*) (SV 4b) and 1042Δ*gttA* (SV 4d, CFSE-stained) before and after enrichment using paramagnetic beads coated with PSA_gp15-GFP.

As A006_gp17 and PSA_gp15 specifically recognize and differentiate SV 1/2 and 4b strains, respectively, we aimed to develop a rapid diagnostic assay for these commonly pathogenic SVs, employing paramagnetic bead-based separation (25). Initially, we coupled GFP-tagged A006_gp17 and PSA_gp15 proteins to activated epoxy beads and used fluorescence microscopy to visualize fluorescent *Listeria* EGDe::pPL2(*rfp*) (SV 1/2) or 1042pPL2(*rfp*) (SV 4b) immobilized on the bead surface (Fig. 3). Although the beads are only 2.8 μm in diameter, the A006_gp17-functionalized beads were highly efficient at cell enrichment, as up to 10 cells could be bound per bead when an excess of bacteria was added (Fig. 3A). We then tested the enrichment efficiencies of both beads from mixed bacterial populations. When A006_gp17 beads were added to a mixed population of EGDe and the rhamnose-deficient mutant EGDeΔ*rmlB* (SV 3a) (*rmlB* is encoded in the rhamnosylation operon, and its deletion leads to loss of rhamnose on the WTA) (14) was prestained with carboxyfluorescein succinimidyl ester (CFSE), the beads specifically bound the target cells, further demonstrating their specificity toward SV 1/2 cells (Fig. 3B). The PSA_gp15 beads offered a similar specificity toward SV 4b cells when added to a mixed population of wild-type 1042 and the galactose-deficient knockout mutant 1042Δ*gttA* (SV 4d) (15) (Fig. 3D). Although PSA_gp15 was highly specific toward the 1042::pPL2(*rfp*) cells, their binding affinity appeared lower than the A006_gp17 beads, as bacteria quickly dissociated from the beads following magnetic enrichment. We assume the weaker binding observed for PSA_gp15 is due to its lower affinity for cell surface receptors than that for the A006_gp17. This reduced binding capacity correlates with the reduced fluorescence signal of PSA_gp15 relative to that of A006_gp17 (Fig. 2A; see Fig. S5 in the supplemental material).

### Improved cell separation by directional coupling of RBPs to the bead surface.

**FIG 4:**
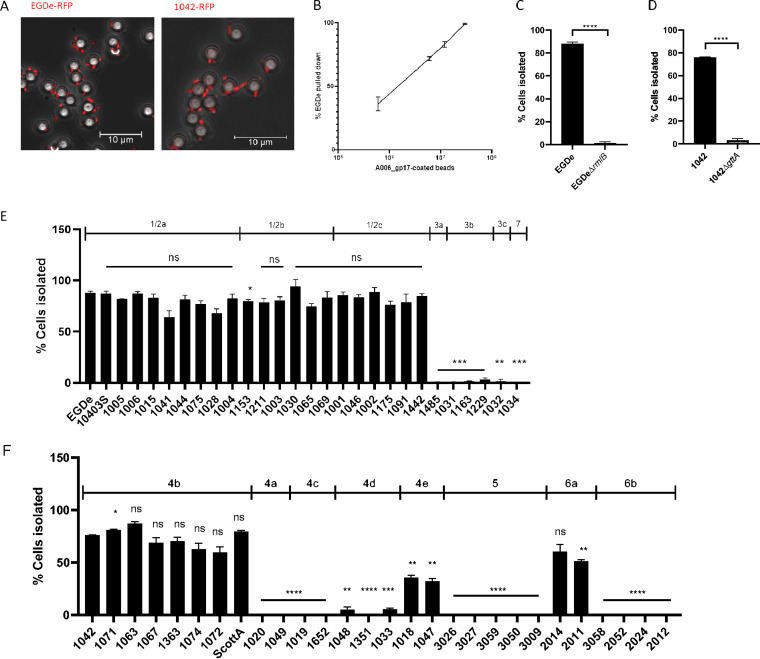
Specific separation of Listeria monocytogenes SV 1/2 and 4b cells using streptavidin-coated magnetic beads conjugated with biotinylated phage RBP protein. (A) EGDe::pPL2(*rfp*) (left) and 1042::pPL2(*rfp*) (right) bound to magnetic beads conjugated with A006_gp17-biotin and PSA_gp15-biotin, respectively, as determined by fluorescence microscopy superimposed on a phase contrast image. (B) Percentage of 10^4^ CFU/ml stationary-phase EGDe cells isolated from a 1-ml reaction volume containing increasing A006_gp17-biotin coated bead numbers. The line on the graph passes through the mean of triplicate experiments with error bars representing SD. (C) Percentage of 10^4^ CFU/ml late-stationary-phase EGDe wild-type and EGDeΔ*rmlB* cells isolated from a 1-ml reaction volume containing 10^7^ beads coated with A006_gp17-biotin. (D) Percentage of 10^4^ CFU/ml late-stationary-phase 1042 wild-type and 1042Δ*gttA* cells isolated from a 1-ml reaction volume containing 10^7^ beads coated with PSA_gp15-biotin. For C and D, mean values ± SD from triplicate experiments are shown; ****, *P* < 0.0001. (E) Percentage of 10^4^ cells isolated from a cohort of L. monocytogenes harboring type I WTAs (with indicated serovar type) using 1 ml of 10^7^ beads coated with A006_gp17-biotin. (F) Percentage of 10^4^ cells isolated from a cohort of L. monocytogenes harboring type II WTAs (with indicated serovar type) using 1 ml of 10^7^ beads coated with PSA_gp15-biotin. For E and F, the percentage pulldown of each strain is statistically compared to EGDe (E) and 1042 (F) as follows: ****, *P* < 0.0001; ***, *P* < 0.001; **, *P* < 0.01; *, *P* < 0.05; ns, no significance.

As epoxy-based conjugation is based on the coupling of primary amino and sulfhydryl groups, the orientation of proteins coupled to the bead surface is random and may render the C-terminal receptor binding (active) sites of the RBPs often inaccessible for interaction with bacterial cells (49–52). This effect could explain the observation that *Listeria* 1042-RFP cells rapidly dissociated from PSA_gp15-coated epoxy beads (Fig. 3D). To address this limitation, we replaced the N-terminal GFP tag of the RBPs with an avidin tag (A006_gp17b and PSA_gp15b), which triggers N-terminal biotinylation by a biotin ligase during recombinant expression in a suitable Escherichia coli strain. Coupling of biotinylated RBPs to streptavidin-coated beads provides directional orientation of the tail spikes, positioning their receptor-binding C termini away from the bead surface, thereby maximizing their exposure and avoiding steric hindrance. With these functionalized beads, separation was highly efficient, yielding pulldown rates of more than 70%, with no observable dissociation (Fig. 4A). In the case of PSA-gp15b-coupled beads, some agglutination was observed, which did not negatively affect separation efficacy (see Fig. S6 in the supplemental material).

To determine optimal conditions for bead-based enrichment, we evaluated the bead-to-cell ratio with the most efficient binding capacity. We found that a 3,000:1 bead-to-cell ratio led to a >99% isolation rate (Fig. 4B), while a 1,000:1 ratio led to a >70% isolation rate for A006_gp17b and PSA_gp15b, which was sufficient for our experiments (Fig. 4C and D). This efficiency is somewhat dependent upon the volume of the mixture; the efficiency of isolation would undoubtedly increase if the overall volume of the bead-bacterium mixture was decreased. Phosphate-buffered saline with Tween 20 (PBS-T; 0.1% Tween 20) was found to be the most effective assay buffer (see Fig. S7A in the supplemental material). Interestingly, recovery rates were lower when using cells at logarithmic-growth phase; yet, a significant difference remained between wild-type and mutant strains (Fig. S7B). Recovery of Gal- and rha-glycosylation mutants (1042Δ*gttA* and EGDeΔ*rmlB*, respectively) was less than 5%, demonstrating a high level of specificity (Fig. 4C and 4D; Fig S7B).

**FIG 5:**
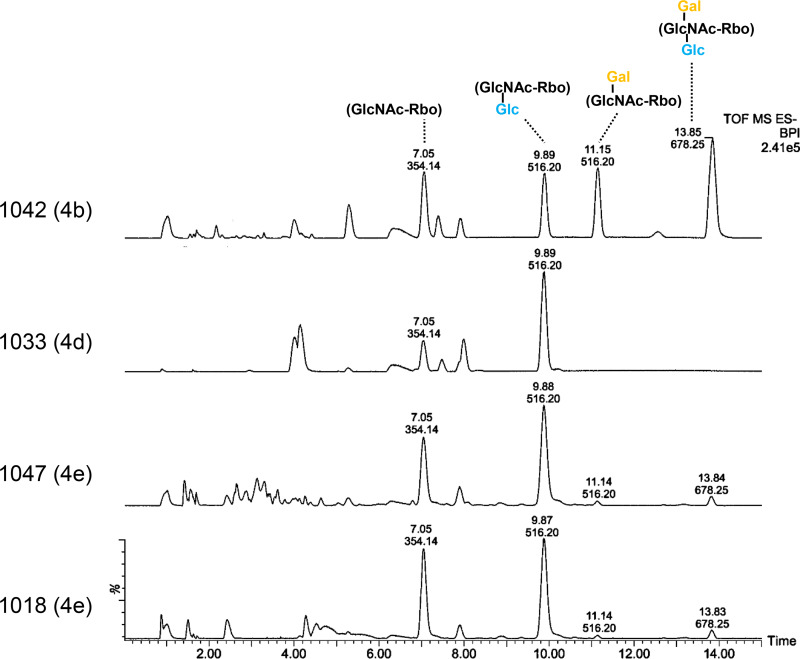
Structural analysis of WTA monomers from designated SV 4e strains. The WTA monomer structures of the indicated strains, as determined by liquid chromatographic separation and MS-based identification (UPLC-MS/MS) and the corresponding serovar designation, are shown here. In the chromatograms, peaks are labeled with their corresponding retention time and relevant peaks are labeled with their assigned structures derived from the *m/z*. The structure of the fully glycosylated species (*m/z*, 678.25) is indicated to the right (Gal, galactose; Glc, glucose; Ac, *O*-acetylation; GlcNAc, *N*-acetylglucosamine).

We then tested the ability to specifically isolate strains of SV 1/2 and 4b. All strains featuring type I WTAs were evaluated against A006_gp17 beads. We observed that SV 1/2 (a, b, and c) strains were recovered at a rate greater than 70%, with only strain 1153 differing from the EGDe control (Fig. 4E). Binding and recovery were negligible for strains of SVs 3 (a, b, and c) and 7. Similarly, the PSA_gp15-biotin-coated beads demonstrated a high rate of pulldown for all 4b strains, although there was some variation in the recovery rates (Fig. 4F). Cells of SV 6a were also bound because their WTA also possesses galactosylated GlcNAc connected to the C4 of Rbo-P (Fig. S2). Curiously, we found that SV 4e strains could also be isolated using PSA_gp15-coated beads, although the efficiency was significantly lower than that observed for SV 4b strains (∼30% versus 70%), suggesting that these strains may feature a low abundance of galactosylated WTA on their cell wall (Fig. 4F). To validate this hypothesis, we extracted WTAs from SV 4e strains 1047 and 1018 and analyzed the repeating unit structure via ultraperformance liquid chromatography-mass spectrometry (UPLC-MS). Our results indicated that these strains, in fact, harbor a tiny trace of 4b-like WTA molecules (Gal-GlcNAc-Glc) identical to the archetypical SV 4b strain 1042 (Fig. 5), explaining why these (rare) strains are distinct from SVs 4b and 4d. As a conclusion, SV 4e strains appear to display an intermediate 4b-4d phenotype. Overall, these results demonstrate the ability of the functionalized magnetic beads developed here to specifically isolate cells of the highly virulent SV 1/2 and 4b, with the caveat that the nonpathogenic SV 6a Listeria innocua and very rare SV 4e L. monocytogenes may be difficult to distinguish from 4b due to their somatic antigen similarity.

## DISCUSSION

Here, we present a novel glycotyping scheme for *Listeria* cells, based entirely on the extraordinary recognition specificity and binding affinity of phage-encoded tail spike proteins and endolysin cell wall-binding domains (CBDs). L. monocytogenes is an opportunistic foodborne pathogen whose virulence correlates with serovars, which are largely represented by the diverse WTA repeating unit structures and their glycosylation patterns (10). Multitudes of differentiation systems exist for *Listeria* beyond classical serotyping, some with great discerning power (5, 49, 53). While standard somatic antigen evaluation is inferior to current genotypic analyses to track and identify outbreaks (50), the correlation between SV and pathogenic potential is ever-present, and rapid analyses are always sought after. Previously used phage typing methods are inexpensive, simple, and relatively quick, but not all isolates can be typed, and the technique lacks discerning power due to the dependence on visual determination of plaque formation and the problems associated with phage resistance PCR-based methods also exist to differentiate SVs 1/2 and 4b strains (49). However, it has been shown that SV changes can result from small, mostly single nucleotide point mutations in glycosyl transferases (14, 15), meaning that SV cannot always be accurately determined by the presence or absence of DNA sequences. Additionally, PCR-based methods do not discriminate for cell viability and can, therefore, produce high rates of false positives due to the mere presence of *Listeria* DNA in food or other samples. This issue also pertains to WGS, which is a relatively slow process. Also, as it has been shown that a serovar change can result from a single loss-of-function SNP (14, 15), it would be nearly impossible using WGS to accurately predict whether a strain is, for example, SV 1/2a versus 3a or 4b versus 4d. Recent developments in core genome multilocus sequence typing (cgMLST) and high-quality SNP (hqSNP) analysis have allowed for modeling of the *Listeria* population structure with an unprecedented level of precision (53–55). However, given the strong correlation between SV and pathogenic potential and the emerging evidence demonstrating the direct effects somatic WTA antigens have on maintaining virulence characteristics and function (15, 17, 56), conceptual advances in rapid phenotypic differentiation are highly desirable.

Fluorescence quantification enables real-time determination of the relative amount of GFP-labeled phage proteins bound to carbohydrate ligands on the bacterial cell surface. This glycotyping approach also allows an estimation of relative amounts of WTA ligand availability within strains of the same SV, leading to significantly greater discerning power than traditional serotyping. Differences in WTA ligand availability can depend on several factors. For one, the absolute amount of WTA subunits can vary greatly, due to differences in polymer chain length (12, 57). In theory, differences in the overall amount of WTA, as well as the portion of WTA extending beyond the surface of the peptidoglycan, could lead to a perceived difference in the quantity of phage protein immobilized to the surface. The direct availability of a ligand can also be dependent upon steric hindrance. For example, CBDP35 binds to GlcNAc residues on WTA polymers (8, 36) but does not bind to SV 4b WTA, despite the presence of GlcNAc. The reason for this is that the GlcNAc in the latter type is not a terminal molecule but is integrated into the chain and also substituted with additional monosaccharides, thereby preventing CBDP35 binding. Hence, the amount of phage protein binding does not always correlate with the amount of ligand present. For this reason, it was necessary to develop a quantitative binding fingerprint for all SVs with the six selected phage proteins (Fig. 2), thereby also eliminating assay bias and subjectivity associated with visual determination by microscopy or agglutination.

SV 4e strains have always been notoriously difficult to identify. In classical serotyping, 4e presents a 4b-like phenotype but may be recognized by antibodies defining SVs 4a and 4d strains (8). Genetically, 4b, 4d, and 4e strains are difficult to distinguish due to their high similarity, and in genotypic differentiation studies, are often clustered together (29, 58). Structurally, the WTA of SV 4e strains has not been fully defined, so we were somewhat intrigued when the 4b-specific pulldown assays yielded an intermediate separation efficiency—roughly half compared to 4b strains. Further analysis of the two SV 4e strains revealed that the majority of the WTA monomer species resembled that of SV 4d, but a minor amount featured the galactose decoration associated with 4b WTA. This suggested that while the majority of the WTA in SV 4b is galactosylated (10), only a very small fraction is galactosylated in SV 4e, thus rendering it an intermediate phenotype between SVs 4b and 4d.

The PSA-gp15 protein used here has a potential limitation since it recognizes both SV 6a and SV 4b, which possess the same galactose decoration and the same GlcNAc position in their WTA. Within L. monocytogenes, the gp15-based signal is entirely specific for SV 4b. However, it is required to determine whether the isolate is L. monocytogenes or *L. innocua*.

The SV 1/2-specific A006_gp17 protein also presents a small limitation. SV 1/2 strains display one of three different H antigens (a, b, and c) (8), and strains presenting the third (SV 1/2c) do not typically cause listeriosis, likely because strains belonging to this particular genetic group harbor an inactivating mutation in the *inlA* virulence factor (59). As glycotyping can distinguish and isolate strains based only on their somatic antigens, a further test for H antigens may be needed to further differentiate strains positively identified using the A006_gp17 protein.

Further developments and applications involving the described differentiation and isolation system remain to be explored. It would be interesting to validate the discerning power of this system in the complex matrices presented by food homogenates or clinical samples. Further improvements could be made by conjugating these proteins to different fluorophores, which will also allow for an application empowered by flow cytometry analysis. Immunomagnetic separation (IMS) using antibodies for the isolation of *Listeria* cells has been published (35, 60–63). However, these systems are hampered by poor recovery rates, lack of specificity, and cross-reactivity. Although several antibodies for L. monocytogenes SV 4b have been reported, they were shown to lack specificity and also bound SV 4a and 4d cells (64, 65). The use of CBD phage proteins conjugated to beads for the selective enrichment of L. monocytogenes from spiked foods has been explored previously, with superior results (34, 35). Given this and the fact that PSA_gp15- and A006_gp17-coated beads retained their specificity in several different buffers with various pH, phage proteins are promising for their use in further applications. Relative to serotyping antibodies, recombinant phage proteins can be produced rapidly and inexpensively, supporting their potential for widespread use. The glycotyping scheme could theoretically also be combined with a *Listeria*-specific reporter bacteriophage (33, 60, 66) that would encompass serovar-specific enrichment followed by single cell-level detection. This would allow circumvention of pre-enrichment procedures and deliver a higher likelihood of excluding the detection of SVs not associated with pathogenicity. Such a system would offer a reasonably specific and very rapid alternative to current genotyping methods and could offer an alternative to diagnostic laboratories which desire a rapid method to determine the likelihood that a contaminating organism harbors pathogenic potential.

## MATERIALS AND METHODS

### Strains, phages, and protein expression constructs.

A large selection of *Listeria* strains, which had previously been serotyped using the standard agglutination method, was chosen from our lab stock collection (Table S1). The external serovar determination was provided by the National Reference Centre for Enteropathogenic Bacteria and *Listeria* (NENT) Switzerland, using a (limited) set of commercially available *Listeria* antisera (Denka Seiken Co. Ltd., Japan), and performed as described (8). All E. coli and genetically modified *Listeria* strains are referenced in Table S2 in the supplemental material. DNA constructs used in this study were cloned and expressed in E. coli XL1-Blue (Stratagene, San Diego, USA) and BL21-GOLD(DE3) (Agilent Technologies, Santa Clara, USA) (see Table S3 in the supplemental material). All *Listeria* strains were grown at 37°C overnight in one-half-strength brain heart infusion (BHI) medium and then were reinoculated to produce log-phase cultures or used straight in the cases where stationary-phase cultures were used. E. coli strains were grown in LB medium with tetracycline (30 μg/ml) and ampicillin (100 μg ml^−1^) for the selection of plasmids. Propagation of bacteriophages was performed as previously described (67, 68). Phage lysates were used as the templates for PCR amplification of the phage proteins in question.

### DNA techniques and cloning procedures.

For the creation of recombinant phage proteins, expression vectors were created using both restriction digestion and Gibson assembly. The pB025_gp18-GFP vector was created by inserting a *gfp* followed by a SacI restriction site between the NdeI and BamHI restriction sites of the pET302 NT-His vector (Invitrogen, Carlsbad, CA, USA); subsequently, a commercially ordered DNA fragment of the full-length B025_gp18 sequence (GeneArt; Thermo Fisher Scientific, Waltham, USA) was inserted between SacI and BamHI, as previously described (36). The pA500_gp19-GFP vector was created by ligating the full-length A500_gp19 sequence (amplified by primers A500_gp19-F/R from whole purified phage) into the SacI-SalI restriction sites of the pHGFP vector (16, 37). The pA006_gp17-biotin vector was created by inserting the truncated A006_gp17 sequence (amplified from whole A006 phage) via Gibson assembly (69) into the p165-His/Avi-tagged vector. Attempts to introduce the N-terminally truncated PSA_gp17 protein into the same p165 vector were unsuccessful. We, therefore, employed a kpOAD tag which employs the biotin ligase expressed natively by E. coli. This led to the viable production of protein, which could successfully be coupled to streptavidin beads. This pHXaKpOAD vector was created by inserting the KpOAD sequence (70) (synthesized as a string) into the StuI and BamHI restriction sites of pQE30-Xa (Qiagen). The pPSA_gp15-biotin vector was created by amplifying the PSA_gp15 N-terminally truncated sequence (see Table S4 in the supplemental material) and assembling with the amplified and linearized pHXaKpOAD via Gibson assembly (New England BioLabs). The pA006_gp17-biotin vector was created in the same fashion but using the p165-His/Avi-tagged vector as a backbone (as previously performed [41]), which expresses biotin ligase (*birA*) downstream of the multiple cloning site (MCS), as previously performed (25). All vectors were confirmed by commercial Sanger sequencing (GATC Biotech AG, Constance, Germany) using primers indicated in Table S4. All other vectors used were previously described (Table S3).

### Expression and purification of fluorescent and biotinylated phage proteins.

Overexpression of recombinant proteins under the control of an isopropyl-β-d-thiogalactopyranoside (IPTG)-inducible promoter was performed using E. coli BL21-GOLD(DE3) in lysogeny broth-protein expression (LB-PE) (22) with 1 mM IPTG to induce expression once growth at 37°C reached an optical density of 0.5. Cultures were further incubated at 19°C overnight, followed by lysis, and then purified on a nickel-affinity column, as previously described (28). Biotinylated proteins were prepared similarly from the constructs indicated in the supplemental tables, except 40 μM biotin was added to the culture at the same time as IPTG. Purity and identity of phage proteins were analyzed by SDS-PAGE using Mini-PROTEAN TGX Stain-Free precast gels (Bio-Rad, Hercules, CA, USA). The final protein solutions were dialyzed twice in a buffer containing 100 mM NaCl, 50 mM NaH_2_PO_4_, and 0.005% Tween 20 (pH 7.4) and then sterile filtered with a 0.2-μm pore filter. Proteins were stored at 4°C.

### Quantitative *Listeria* phage protein-based cell wall-binding assays.

A graphical summary of the *Listeria* phage protein-based cell wall-binding assays is shown in Fig. 1A. *Listeria* strains in log phase (OD_600_, 0.8) were harvested by centrifugation (7,000 × *g*, 10 min, 4°C) and resuspended in 1/10 of their initial culture volume of PBS-T (0.2 g/liter KCl, 0.2 g/liter KH_2_PO_4_, 8 g/liter NaCl, 2.16 g/liter Na_2_HPO_4_·7H_2_O, and 0.05 % Tween 20). A mixture of 100 μl of bacterial suspension and 50 μl of protein at a concentration of 0.25 mg/ml was incubated at room temperature RT for 30 minutes, with shaking at 150 rpm. A control containing only GFP was included in all assays. Cells were then harvested by centrifugation, washed once with 1 ml PBS-T, and resuspended in 150 μl PBS-T. For fluorescence quantification, the OD was measured, suspensions were transferred to a black polystyrene 96-well plate (Nunc; Thermo Fisher Scientific, Waltham, USA), and fluorescence was quantified with a Fluostar Omega plate reader (BMG Labtech, Ortenberg, Germany). Signals were normalized to the measured OD, and the fluorescence of the GFP control was subtracted from the fluorescence of all other proteins for each strain.

### Fluorescence microscopy.

For microscopy, 5 μl of *Listeria* suspensions labeled with fluorescent proteins as described above or 5 μl of *Listeria* coupled to paramagnetic beads were transferred onto a glass slide with a cover slip. Imaging was performed on a TCS SPE confocal system (Leica Microsystems GmbH, Germany) equipped with an HCX PL Fluotar 100 × 1.30 oil objective. GFP and CFSE were excited at λ of 488 nm, and red fluorescent protein (RFP) was excited at λ of 532 nm. For 3D reconstructions, z-stacks of 20 μm with a z-slice thickness of 0.5 μm were imaged. Image analysis was performed in Imaris Software (Bitplane AG, Zurich, Switzerland), and for ease of visualization, the contrast in red and green channels was enhanced.

### Coupling of proteins to paramagnetic beads.

Coupling of A006_gp17-GFP and PSA_gp15-GFP to epoxy-coated beads (Dynabeads M-270 Epoxy; Thermo Fisher) was performed according to the manufacturer’s protocol. Briefly, 1.5 mg of beads (corresponding to ∼10^8^ beads) were resuspended and washed twice (each time using a magnet to pull down beads) in 100 mM sodium phosphate buffer (pH 7.4). Beads were resuspended in a final volume of 100 μl of sodium phosphate buffer, to which 100 μl of fluorescent protein solution (concentration adjusted to 1 mg/ml) was added along with 100 μl of 3 M (NH_4_)_2_SO_4_. Coupling was performed overnight at 37°C under constant rotation. Following incubation, beads were washed four times with PBS (pH 7.4) before being used for L. monocytogenes pulldown. The efficiency of the protein coupling was evaluated by fluorescence microscopy as described above.

Coupling of biotinylated proteins to streptavidin beads (Dynabeads M-280 Streptavidin; Thermo Fisher) was also performed according to the manufacturer’s protocol. Briefly, for each pulldown, the volume of beads was calculated from a concentration of 7 × 10^8^ beads/ml. Beads were washed in PBS-T (0.1% Tween 20) before being resuspended in a protein solution containing 1 mg biotinylated protein (A006_gp17-biotin or PSA_gp15-biotin) per ml of beads (1 mg for 7 × 10^8^ beads). The beads were incubated at RT for 30 min on an overhead rotator before being washed with PBS-T four times. The beads were then used immediately for subsequent pulldown experiments.

### Paramagnetic bead-based separation.

For the retrieval of L. monocytogenes using fluorescent proteins coupled to epoxy-coated beads, RFP-expressing WT L. monocytogenes strains EGDe::pPL2(*rfp*) (71) and 1042::pPL2(*rfp*) (this study) were used, as well as their WTA glycosylation-deficient deletion mutants (1042Δ*gttA* and EGDeΔ*rmlB*) (14, 15). Overnight cultures of mutants were stained with the green fluorescent membrane stain CFSE as follows: 1 ml of a culture at OD 0.5 to 0.7 was spun down and washed with PBS-T. CFSE was added to a final concentration of 5 μM in a total volume of 1 ml PBS-T to the cells, followed by incubation at 37°C for 20 min and four subsequent washes (using a magnet to pull down beads) with PBS-T, followed by resuspension in the original volume of PBS-T. To evaluate epoxy bead retrieval specificity, WT-RFP and CFSE-stained mutant bacteria were mixed 1:1 (200 μl each, along with 10 μl of beads—see above). The bead-bacterium mixture was incubated at RT for 30 min on an overhead rotator before being washed twice with PBS-T and resuspended in a final volume of 50 μl PBS-T. The serovar pulldown specificity of this mixture was examined by confocal microscopy as described above. Evaluation of L. monocytogenes binding to biotinylated protein-coated beads (A006_gp17-biotin or PSA_gp15-biotin) was also performed using confocal microscopy to visualize RFP-expressing 1042 or EGDe.

To calculate recovery rates, biotinylated proteins (A006_gp17-biotin or PSA_gp15-biotin) coupled to streptavidin beads were utilized along with an automatic bead retriever (Applied Biosystems). Single strains of L. monocytogenes at stationary phase (unless otherwise indicated) were washed with PBS-T, and 10^4^ cells were employed for each individual pulldown along with the coated beads (10^7^ beads unless otherwise specified) in a total volume of 1 ml PBS-T. Mixtures were incubated for 30 min with constant agitation, followed by magnetic separation and two washes in 1 ml PBS-T, followed by resuspension of the beads in 1 ml PBS-T. The final suspension as well as the supernatant (cells left over in the original well) were enumerated by plating on one-half-strength BHI agar. The fraction of L. monocytogenes isolated was calculated by dividing the CFU isolated by the sum of the CFU isolated and the CFU left over in the supernatant.

### WTA extraction and mass spectrometry of SV 4e strains.

Cell wall purifications of strains 1042, 1047, and 1018 were performed as previously described (72). Structural determination was performed as previously described using a UPLC-tandem MS (MS/MS) method (10). The relative amounts of galactosylated species were examined by comparing the area under the peaks within each chromatogram. Major peaks were assigned based on previous analysis of strain 1042.

### Statistics.

To evaluate statistical significance, a Student’s *t* test or a one-way paired analysis of variance (ANOVA) followed by a multiple comparison was employed, as indicated in the figure legends.

## Supplementary Material

Supplemental file 1
