# Supplementary material for: Glycotyping and Specific Separation of Listeria monocytogenes with a Novel Bacteriophage Protein Tool Kit
Source: Appl Environ Microbiol. 2020 Jun 17;86(13):e00612-20. doi: 10.1128/AEM.00612-20 (PMC7301860; doi:10.1128/AEM.00612-20)
Supplement: Supplemental file 1 [file AEM.00612-20-s0001.pdf]

Supplemental Information (Sumrall et al.)

Suppl. Table S1. Sixty *Listeria* strains tested with the quantitative *Listeria* phage protein based cell-wall binding assays. The previously determined (and published, if applicable) serovar is listed, as well as the glycotype determined in this study.

Species	WSLC[a] code	Source	Serovar	Glycotype	
L. monocytogenes	EGDe	Ref. (1)	1/2a	1/2	*
L. monocytogenes	10403S	Daniel Portnoy	1/2a	1/2	
L. monocytogenes	1005	SLCC[b] 5634	1/2a	1/2	
L. monocytogenes	1004	SLCC 5633	1/2a	1/2	
L. monocytogenes	1041	ATCC[c] 19111	1/2a	1/2	
L. monocytogenes	1006	NCTC 7973	1/2a	1/2	
L. monocytogenes	1015	SLCC 5635	1/2a	1/2	
L. monocytogenes	1044	SLCC 5764	1/2a	1/2	
L. monocytogenes	1028	SLCC 5758	1/2a	1/2	
L. monocytogenes	1153	Cheese	1/2a	1/2	
L. monocytogenes	1211	Cheese	1/2a	1/2	
L. monocytogenes	1004	SLCC 5633	1/2a	1/2	
L. monocytogenes	1030	SLCC 2755	1/2b	1/2	
L. monocytogenes	1075	SLCC 8811	1/2b	1/2	
L. monocytogenes	1030	SLCC 2755	1/2b	1/2	
L. monocytogenes	1065	SLCC 8797	1/2b	1/2	
L. monocytogenes	1069	SLCC 8798	1/2b	1/2	
L. monocytogenes	1003	SLCC 2375, ATCC 19115	1/2b	1/2	
L. monocytogenes	1001	ATCC 19112	1/2c	1/2	
L. monocytogenes	1091	SLCC 8848	1/2c	1/2	
L. monocytogenes	1046	SLCC 1807	1/2c	1/2	
L. monocytogenes	1175	Cheese	1/2c	1/2	
L. monocytogenes	1442	Ref. (2)	1/2*	1/2	
L. monocytogenes	1485	Cheese	3a	3	*
L. monocytogenes	1031	SLCC1694	3b	3	
L. monocytogenes	1163	Cheese	3b	3	
L. monocytogenes	1229	Cheese	3b	3	
L. monocytogenes	1032	SLCC 2479	3c	3	
L. monocytogenes	1034	SLCC 2482	7	7	*

L. monocytogenes	1049	SLCC 2671	4a	4a	
L. monocytogenes	1020	ATCC 19114	4a	4a	*
L. monocytogenes	1351	Chicken	4a	4a	
L. monocytogenes	1042	ATCC 23074	4b	4b	*
L. monocytogenes	1072	SLCC 8807	4b	4b	
L. monocytogenes	1074	SLCC 5653	4b	4b	
L. monocytogenes	ScottA	Ref. (3)	4b	4b	
L. monocytogenes	1363	Cheese	4b	4b	
L. monocytogenes	1063	SLCC 8793	4b	4b	
L. monocytogenes	1071	SLCC 7892	4b	4b	
L. monocytogenes	1067	SLCC 8802	4b	4b	
L. monocytogenes	1019	ATCC 19116	4c	4c	*
L. monocytogenes	1652	Lab Collection	4c	4c	
L. monocytogenes	1039	ATCC 13932	4b	4d or 4e	#
L. monocytogenes	1047	SLCC 1090	4d	4d or 4e	
L. monocytogenes	1033	ATCC 19117	4d	4d or 4e	*
L. monocytogenes	1045	SLCC 1806	4d	4d or 4e	
L. monocytogenes	1048	SLCC 1652	4d	4d or 4e	
L. monocytogenes	1018	ATCC 19118	4e	4d or 4e	*
L. ivanovii	3027	SLCC 4713	5	5	
L. ivanovii	3050	SLCC 4719	5	5	
L. ivanovii	3058	SLCC 3584	5	5	
L. ivanovii	3026	SLCC 5378	5	5	
L. ivanovii	3059	SLCC 3706	5	5	
L. ivanovii	3009	SLCC 4769	5	5	*
L. innocua	2014	Lab Collection	6a	6a	
L. innocua	2011	ATCC 33090	6a	6a	*
L. innocua	2012	ATCC 33091	6b	6b	*
L. innocua	2024	SLCC 5642	6b	6b	
L. innocua	2052	SLCC 4276	6b	6b	

[a] *WSLC (Weihenstephan Listeria Collection) code is used for all strains, except EGDe, 10403S and ScottA*

[b] *SLCC (Special Listeria Culture Collection)*

[c] *ATCC (American Type Culture Collection)*

** Indicates strains with WTA structure verified by mass-spectrometry*

# indicates published strains with unexpected SVs

Suppl. Table S2. *E. coli* and *Listeria monocytogenes* strains used in this study, their source, and original serovar designation before confirmation using the glycotyping system.

Species	Strain	Source	Serovar
E. coli	XL-1 blue	Stratagene	-
E. coli	BL21 (DE3)	Invitrogen	-
L. monocytogenes	EGDe::pPL2(rfp)	Ref. (4)	1/2a
L. monocytogenes	1042::pPL2(rfp)	Ref. (5)	4b
L. monocytogenes	EGDeΔ*rmlB*	Ref. (6)	3a
L. monocytogenes	1042Δ*gttA*	Ref. (5)	4d

Suppl. Table S3. Constructs used for production of the phage-derived proteins used in this study.

Constructs	Description	Source
pQE30	Enables N-terminal fusion with 12-amino-acid leader containing a 6x-His tag, AmpR	Qiagen
pHGFP	gfp-mut2 cloned into BamHI-SacI sites of pQE30	Ref. (7)
pA006_gp17-GFP	N-terminally truncated gp17-encoded RBP (aa345-aa721) from phage A006 cloned into pETDUET-GFP (containing N-terminal GFP) via gibson assembly	This study
pA500_gp19-GFP	Full-length gp19-encoded RBP from phage A500 cloned into SacI-SalI sites of pHGFP	Ref. (8)
pPSA_gp15-GFP	N-terminally truncated gp15-encoded RBP (aa200-aa374) from phage PSA cloned into pHGFP via gibson assembly	Ref. (9)
pET302 NT-His	N-terminally 6x histidine tagged vector for protein purification	Invitrogen
pB025_gp18-GFP	GFP cloned into the Xho1 and Sac1 restriction sites with the full length gp18-encoded RBP cloned downstream into the Sac1-BamH1 restriction sites	This study
pHGFP-CBD500	ply500 fragment encoding aa133-aa289 cloned into SacI-SalI sites of pHGFP	Ref. (10)
pHGFP_CBD025-B	ply025 fragment encoding aa132-aa276 cloned into SacI-SalI sites of pHGFP	Ref. (10)
pHGFP_CBDP35-B	plyP35 fragment encoding aa150-aa291 cloned into SacI-SalI sites of pHGFP	Ref. (10)
p165-His/Avi-tagged	His-avitagged S16LTF with BirA in MCS2.	Ref. (11)
pHXaKpOAD	kPOAD tag (Stolz et al., 1998) ligated into pQE30	This study
pA006_gp17-biotin	N-terminally truncated gp17-encoded RBP (aa345-721) from phage a006 cloned into p165-His/Avi-tag via gibson assembly	This study
pPSA_gp15-biotin	N-terminally truncated gp15-encoded RBP (aa200-aa374) from phage PSA cloned into pHXaKpOAD via gibson assembly downstream of KpOAD tag	This study

Suppl. Table S4. Primers used in this study

Primers	Purpose	Sequence (5'-3')
400	Amplification (F) of the pETDUET-GFP vector for purposes of Gibson assembly to insert the A006_gp17 full-length sequence	ATGGATGAACTATACAAAGAGCTCATGTTATTGATATTAGATGAAAAT
401		ATTTTCATCTAATATCAATAACATGAGCTCTTTGTATAGTTCATCCAT
402 V2	Amplification (F) of the pETDUET-GFP vector for purposes of Gibson assembly to insert the A006_gp17 truncated sequence	TCAATTATTTTATGTTTTAGATTAACTGCAGGTCGACAAGCTTGCGGC
403 P2	Amplification (F and R) of the truncated A006_gp17 for insertion into pETDUET-GFP	GCCGCAAGCTTGTCGACCTGCAGTTAATCTAAAACATAAAATAATTGA
404 P1		ATGGATGAACTATACAAAGAGCTCATGTCAAAAAGCACTTGTGT
405 V1	Amplification (R) of the pETDUET-GFP vector for purposes of Gibson assembly to insert the A006_gp17 truncated sequence	ACACAAGTGCTTTTTGACATGAGCTCTTTGTATAGTTCATCCAT
A500_gp17-F	F and R primers for amplification of the gp19 sequence from phage A500 for SacI/SalI insertion into pHGFP	TTTGAGCTCATGGATTTAAAAAAATGGCAAGACCCA
A500_gp17-R		TTTGTCGACTCATGTTCTAACCACTCCCC
A006 gp17_F2	F and R primers for amplification of the gp17 sequence from phage A006 for	GGCACGAAGTCGAGCTCATGTCAAAAAGCACTTGTGTAGTTGGTG

A006 gp17_R2	insertion into pETDUET-GFP via Gibson assembly	GCAAGCTTGTCGACCTGCAGTTAATCTAAAACATAAAATAATTGATTTAACGCAAAATAAGT
pETDUET1_Upstream	F and R primers for amplification of the pETDUET-GFP vector for purposes of Gibson assembly	ATGCGTCCGGCGTAG A
pETDUET1_down1		GATTATGCGGCCGTGTACAA
pQE30-PSA_fwd	F and R primers for amplification of the pHXaKpOAD vector for purposes of insertion via Gibson assembly of the truncated PSA_gp15 sequence	AATCCCGCTCAATTCGCAC
pQE30-PSA_rev		AGTTAATTTCTCCTCTTTAATGAATTCTGTG
Fritz Tag(psa)_fwd	F and R primers for amplificatin of the truncated PSA_gp15 sequence from the phage PSA genome for purposes of insertion into pHXaKpOAD tp produce a biotinylated product	ACAGAATTCATTAAAGAGGAGAAATTAACTAGGAGAAATTAACTATGAGAGGATC
Fritz Tag(psa)_rev		AACAGTTTTTTGTGCGAATTGAGCGGGATTGGATCCCAGGGTCATCAG

Supplemental references:

1. Vazquez-Boland JA, Kuhn M, Berche P, Chakraborty T, Dominguez-Bernal G, Goebel W, Gonzalez-Zorn B, Wehland J, Kreft J. 2001. Listeria pathogenesis and molecular virulence determinants. Clin Microbiol Rev 14:584-640.
2. Wendlinger G, Loessner MJ, Scherer S. 1996. Bacteriophage receptors on Listeria monocytogenes cells are the N-acetylglucosamine and rhamnose substituents of teichoic acids or the peptidoglycan itself. Microbiology 142 (Pt 4):985-92.
3. Briers Y, Klumpp J, Schuppler M, Loessner MJ. 2011. Genome sequence of Listeria monocytogenes Scott A, a clinical isolate from a food-borne listeriosis outbreak. J Bacteriol 193:4284-5.
4. Studer P, Staubli T, Wieser N, Wolf P, Schuppler M, Loessner MJ. 2016. Proliferation of Listeria monocytogenes L-form cells by formation of internal and external vesicles. Nat Commun 7:13631.
5. Sumrall ET, Shen Y, Keller AP, Rismondo J, Pavlou M, Eugster MR, Boulos S, Disson O, Thouvenot P, Kilcher S, Wollscheid B, Cabanes D, Lecuit M, Grundling A, Loessner MJ. 2019. Phage resistance at the cost of virulence: Listeria monocytogenes serovar 4b requires galactosylated teichoic acids for InlB-mediated invasion. PLoS Pathog 15:e1008032.
6. Eugster MR, Morax LS, Huls VJ, Huwiler SG, Leclercq A, Lecuit M, Loessner MJ. 2015. Bacteriophage predation promotes serovar diversification in Listeria monocytogenes. Mol Microbiol 97:33-46.
7. Loessner MJ, Kramer K, Ebel F, Scherer S. 2002. C-terminal domains of Listeria monocytogenes bacteriophage murein hydrolases determine specific recognition and high-affinity binding to bacterial cell wall carbohydrates. Mol Microbiol 44:335-49.
8. Sumrall E, Schefer C, Rismondo J, Boulos S, Gründling A, Loessner MJ, Y S. 2020. Galactosylated wall-teichoic acid, but not lipoteichoic acid, retains InlB on the surface of serovar 4b Listeria monocytogenes. Mol Microbiol 113:638-649.

9. Dunne M, Rupf B, Tala M, Qabrati X, Ernst P, Shen Y, Sumrall E, Heeb L, Pluckthun A, Loessner MJ, Kilcher S. 2019. Reprogramming Bacteriophage Host Range through Structure-Guided Design of Chimeric Receptor Binding Proteins. Cell Rep 29:1336-1350 e4.

10. Schmelcher M, Shabarova T, Eugster MR, Eichenseher F, Tchang VS, Banz M, Loessner MJ. 2010. Rapid multiplex detection and differentiation of Listeria cells by use of fluorescent phage endolysin cell wall binding domains. Appl Environ Microbiol 76:5745-56.

11. Denyes JM, Dunne M, Steiner S, Mittelviefhaus M, Weiss A, Schmidt H, Klumpp J, Loessner MJ. 2017. Modified Bacteriophage S16 Long Tail Fiber Proteins for Rapid and Specific Immobilization and Detection of Salmonella Cells. Appl Environ Microbiol 83:e00277-17.

12. Shen Y, Boulos S, Sumrall E, Gerber B, Julian-Rodero A, Eugster MR, Fieseler L, Nystrom L, Ebert MO, Loessner MJ. 2017. Structural and functional diversity in Listeria cell wall teichoic acids. J Biol Chem 292:17832-17844.

Suppl. Figure S1. SDS-PAGE of GFP-tagged phage proteins. Expressed and purified proteins (4 µg) were loaded to confirm protein identity and purity; molecular weights of all proteins and the marker are indicated.

Suppl. Figure S2. WTA structures of the *Listeria* genus. WTA monomer structures from representative strains of each serovar (indicated in parentheses), as determined by UPLC-MS/MS. Structures are provided as a reference for understanding binding patterns of the individual phage-derived proteins. The corresponding shapes are explained in the legend. This figure is adapted from reference 12 (© American Society for Biochemistry and Molecular Biology).

Suppl. Figure S3. Fluorescence microscopy of *Listeria* cells labeled with GFP-tagged phage proteins. Images of the indicated strains and their corresponding serovar in parentheses, all labeled with the indicated GFP-tagged proteins. All indicated strains have known WTA structures, and were used as standards (see **(10)**).

Suppl. Figure S4. Quantitative comparison of cell-wall binding of GFP-tagged A006_gp17 and its N-terminal truncation. Mean relative fluorescence intensity of the indicated GFP-tagged proteins expressed as a percentage compared to the EGDe control (truncated A006_gp17; which represents the maximum observed value). Error bars represent the standard deviation.

Suppl. Figure S5. Quantitative determination of GFP-tagged PSA_gp15 binding to *Listeria* strains representing different serovars. Fluorescence-based quantification of binding of PSA_gp15 to different *Listeria* strains (strain name in parentheses) and *S. aureus* Newman as a negative control. The dotted line represents the cutoff value above which the mean fluorescence for a given strain indicates positive binding. Measured fluorescence (Y-axis) is presented as arbitrary fluorescence units. Measurements were performed in quadruplicates; error bars represent standard errors of the mean (S.E.M.).

Suppl. Figure S6. Evaluating the specificity and effectiveness of PSA_gp15-biotin coupled to streptavidin beads. A 1:1 mixture of 1042::pPL2(*rfp*) (SV 4b, red) and 1042Δ*1095* (SV 4d, CFSE-stained, green) at stationary phase, before and after magnetic separation with

streptavidin beads coated with PSA_gp15-biotin. Bacteria were mixed with beads being in excess. Agglutination was frequently observed using these beads with strain 1042.

Suppl. Figure S7. Pulldown rates in varying buffers and specificity tests for *Lm* at logarithmic growth phase. A) Pulldown rates of 10^4 1042 cells at stationary growth phase by 10^7 PSA_gp15-biotin-coated beads mixed, washed and eluted in the four indicated buffers (representation is mean ± SD of three experiments). B) Pulldown rate of 10^4 cells at logarithmic growth phase of the indicated strains individually mixed with 10^7 beads coated with A006_gp17-biotin (left) or PSA_gp15-biotin (right) in 1 mL PBS-T (representation is mean ± SD of three experiments; ***P<0.001, **P<0.01).

Figure S1

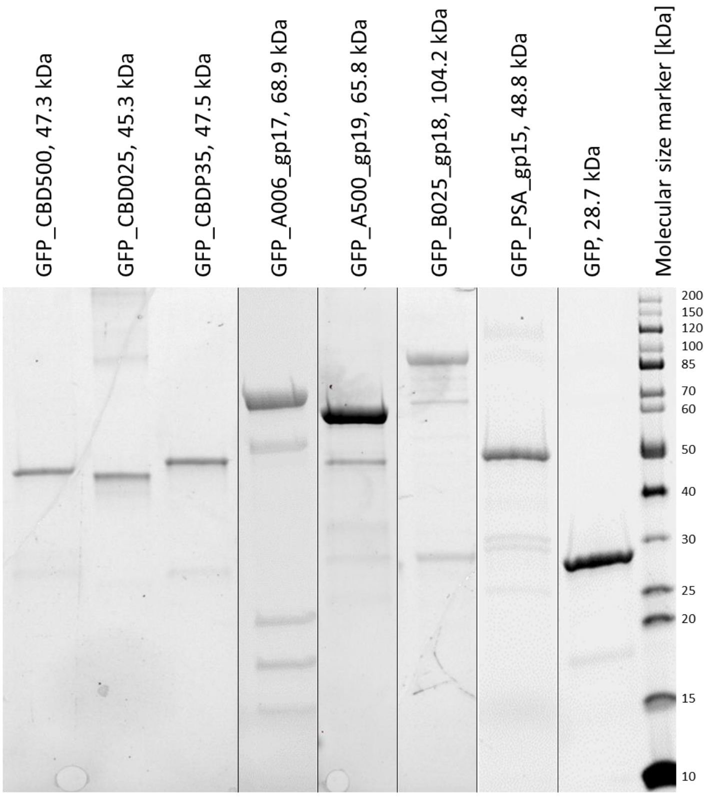

GFP_CBD500, 47.3 kDa
GFP_CBD025, 45.3 kDa
GFP_CBDP35, 47.5 kDa
GFP_A006_gp17, 68.9 kDa
GFP_A500_gp19, 65.8 kDa
GFP_B025_gp18, 104.2 kDa
GFP_PSA_gp15, 48.8 kDa
GFP, 28.7 kDa
Molecular size marker [kDa]
200
150
120
100
85
70
60
50
40
30
25
20
15
10

Figure S2

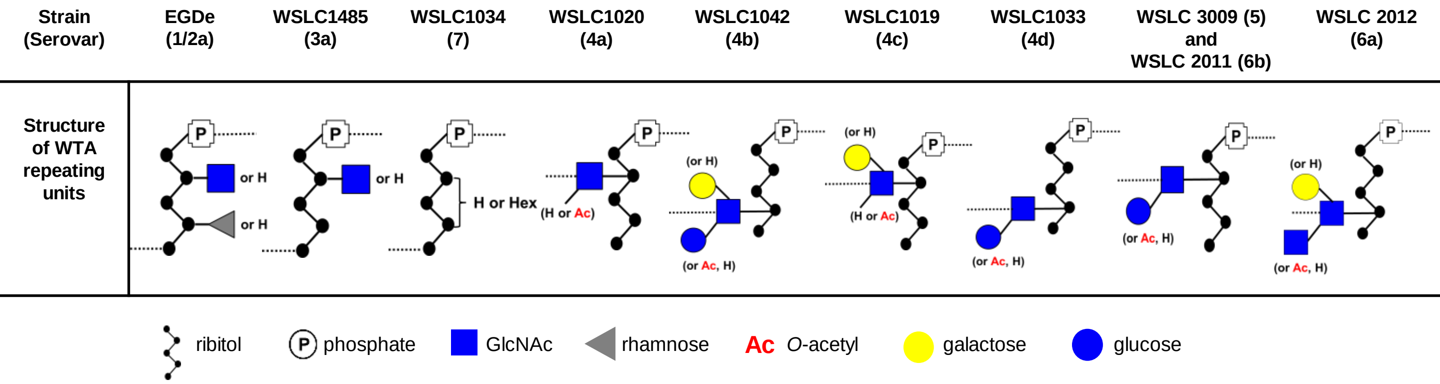

Strain (Serovar)
EGDe (1/2a)
WSLC1485 (3a)
WSLC1034 (7)
WSLC1020 (4a)
WSLC1042 (4b)
WSLC1019 (4c)
WSLC1033 (4d)
WSLC 3009 (5) and WSLC 2011 (6b)
WSLC 2012 (6a)
Structure of WTA repeating units
P
or H
H or Hex
(H or Ac)
(or H)
(or Ac, H)
ribitol
P phosphate
GlcNAc
rhamnose
Ac O-acetyl
galactose
glucose

Figure S3

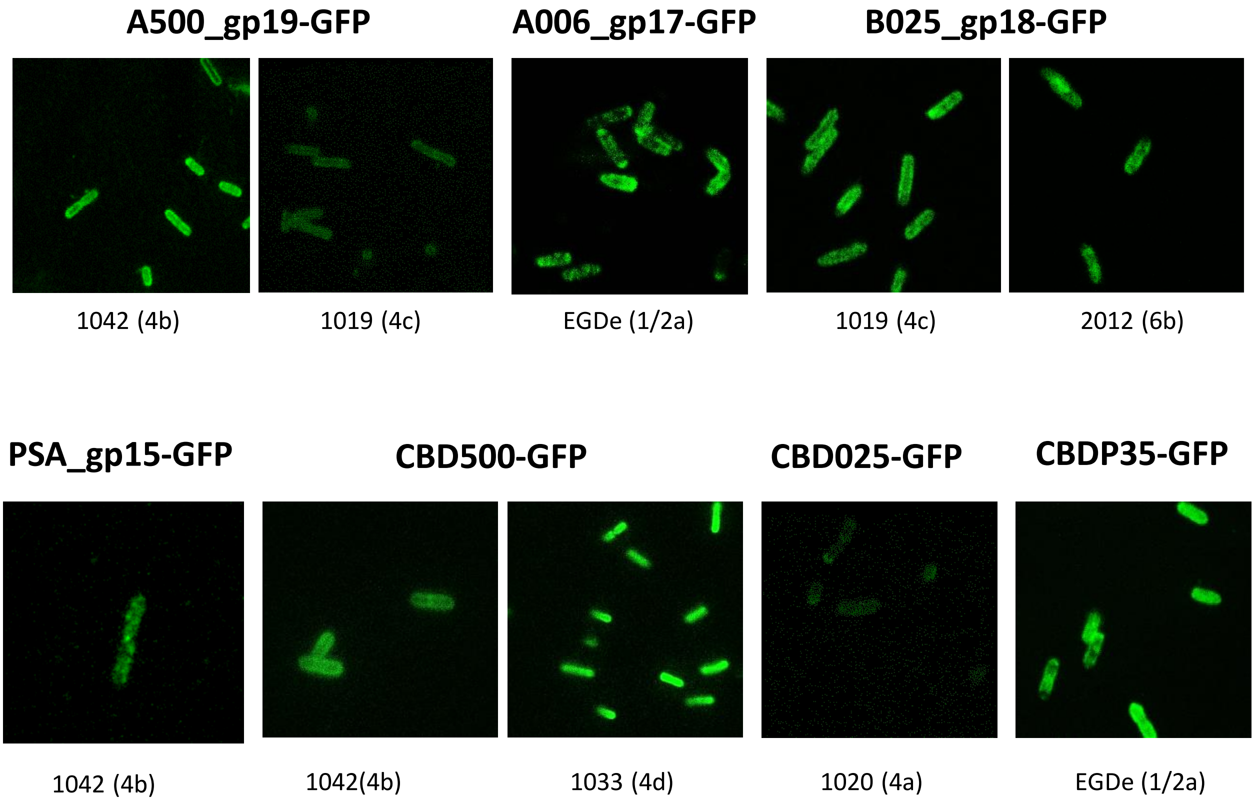
A500_gp19-GFP
A006_gp17-GFP
B025_gp18-GFP
1042 (4b)
1019 (4c)
EGDe (1/2a)
1019 (4c)
2012 (6b)
PSA_gp15-GFP
CBD500-GFP
CBD025-GFP
CBDP35-GFP
1042 (4b)
1042(4b)
1033 (4d)
1020 (4a)
EGDe (1/2a)

Figure S4

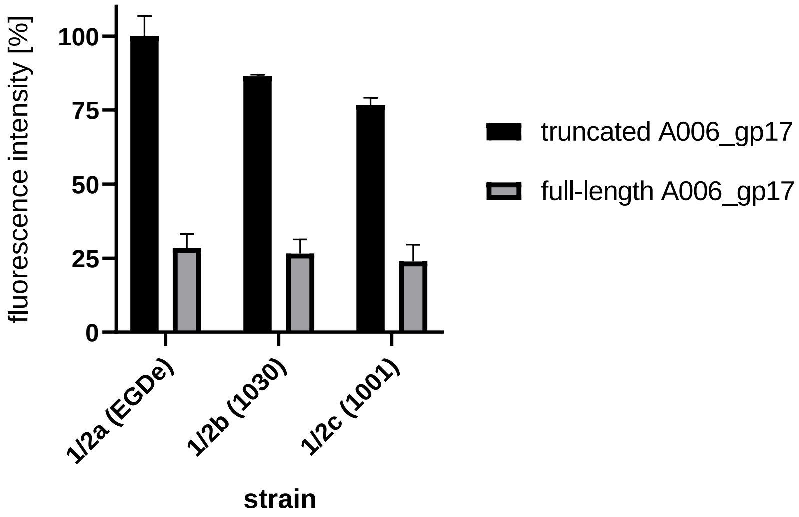

fluorescence intensity [%]
100
75
50
25
0
1/2a (EGDe)
1/2b (1030)
1/2c (1001)
strain
truncated A006_gp17
full-length A006_gp17

Figure S5

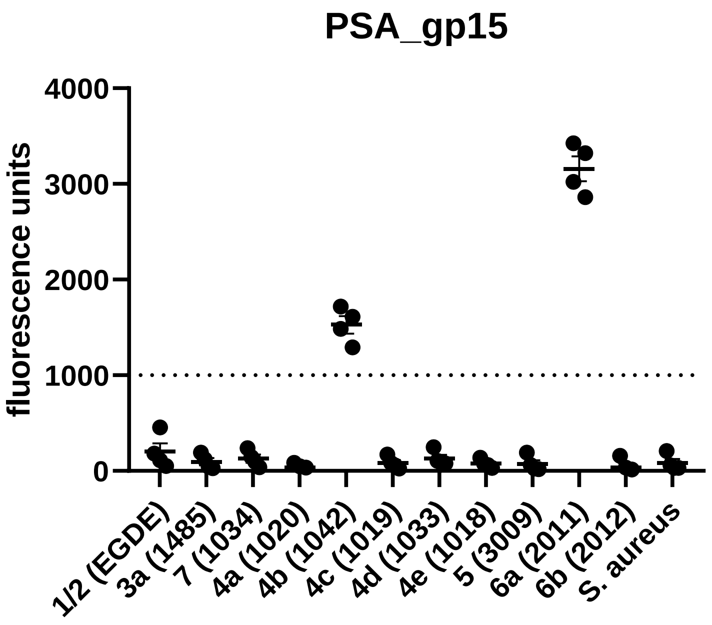
PSA_gp15
fluorescence units
4000
3000
2000
1000
0
1/2 (EGDE)
3a (1485)
7 (1034)
4a (1020)
4b (1042)
4c (1019)
4d (1033)
4e (1018)
5 (3009)
6a (2011)
6b (2012)
S. aureus

Figure S6

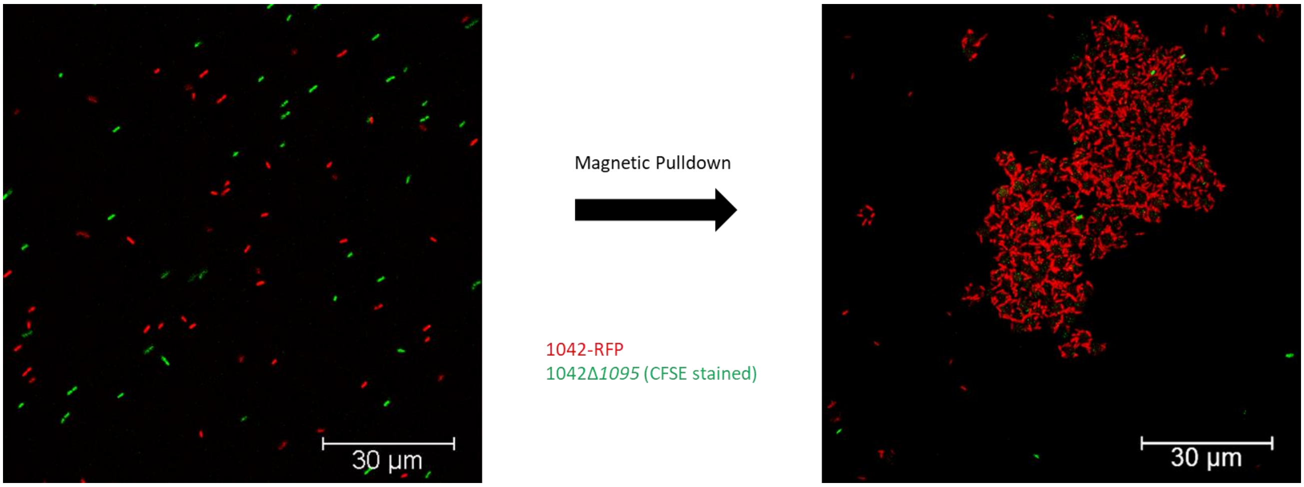
30 µm
Magnetic Pulldown
1042-RFP
1042Δ*1095* (CFSE stained)
30 µm

Figure S7

A

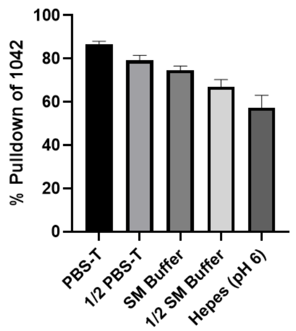

% Pulldown of 1042
100
80
60
40
20
0
PBS-T
1/2 PBS-T
SM Buffer
1/2 SM Buffer
Hepes (pH 6)

B

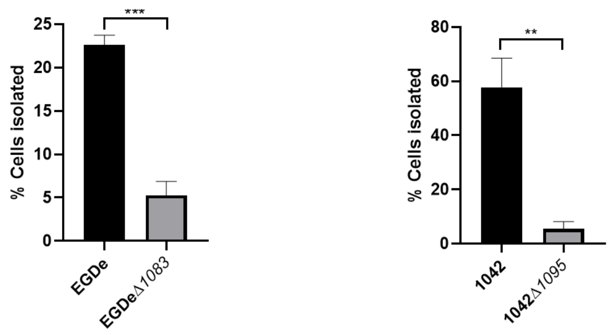

% Cells isolated
25
20
15
10
5
0
***
EGDe
EGDe∆1083
% Cells isolated
80
60
40
20
0
**
1042
1042∆1095
